# Meningocèle temporale et anophtalmie: à propos d´un cas

**DOI:** 10.11604/pamj.2020.37.8.24930

**Published:** 2020-09-02

**Authors:** Patrick Rakotozanany, Miraulle Tien Yu Song, Njara Francia Ranoasy, Ketsia Rakotovao, Willy Ratovondrainy

**Affiliations:** 1 Service de Neurochirurgie, Centre Hospitalier de Soavinandriana (CENHOSOA), Antananarivo, Madagascar,; 2 Service d’Ophtalmologie, CHU/JRA Ampefiloha, Antananarivo, Madagascar

**Keywords:** Anophtalmie, méningocèle, os temporal, Anophthalmia, meningocele, temporal bone

## Abstract

Les auteurs rapportent un cas d´un garcon de 12 mois pour une polymalformation congénitale à type de méningocèle temporale droite et une aplasie du globe oculaire homolatérale qui était présent depuis sa naissance. Le scanner cérébral confirmait la malformation avec un défect osseux au niveau temporal droit, une hernie de la méninge contenant du liquide cérébro-spinal et une absence du globe oculaire droit. La chirurgie était pratiquée pour la méningocèle. L´évolution était favorable. Notre objectif est de montrer la rareté de cette affection à la lumière d´une revue de la littérature.

## Abstract (en)

We here report the case of a 12-month old infant with congenital polymalformation including right temporal meningocele and homolateral eyeball aplasia. Brain CT scan confirmed this malformation with bone defect in the right temporal lobe, meningeal hernia containing cerebrospinal fluid and absence of the right eyeball. Surgery was performed to treat meningocele. Patient’s outcome was favorable. The purpose of this study was to highlight the rarity of this disease on the basis of a literature review.

## Introduction

Les méningocèles sont des malformations congénitales caractérisées par une anomalie du tube neurale avec une hernie des méninges contenant du liquide céphalo-rachidien (LCR). Et les méningo-encéphalocèles contiennent des tissus cérébraux et du liquide céphalo-rachidien (LCR) [1]. L´incidence, en occident, est estimée à 1/35 000 naissances siégeant le plus souvent au niveau de l´étage antérieur de la base du crâne. Ces chiffres sont six fois plus élevés en Asie du Sud-ouest [2, 3]. Sur le plan embryologique, ils sont secondaires à un défaut de fermeture du tube neural au cours de la 4^e^semaine de la gestation. Le scanner cérébral est indispensable pour le diagnostic. Le traitement est basé sur la chirurgie réparatrice [4]. Nous rapportons un cas d´une forme temporale droite associée à une anophtalmie homolatérale.

## Patient et observation

**Figure 1:**
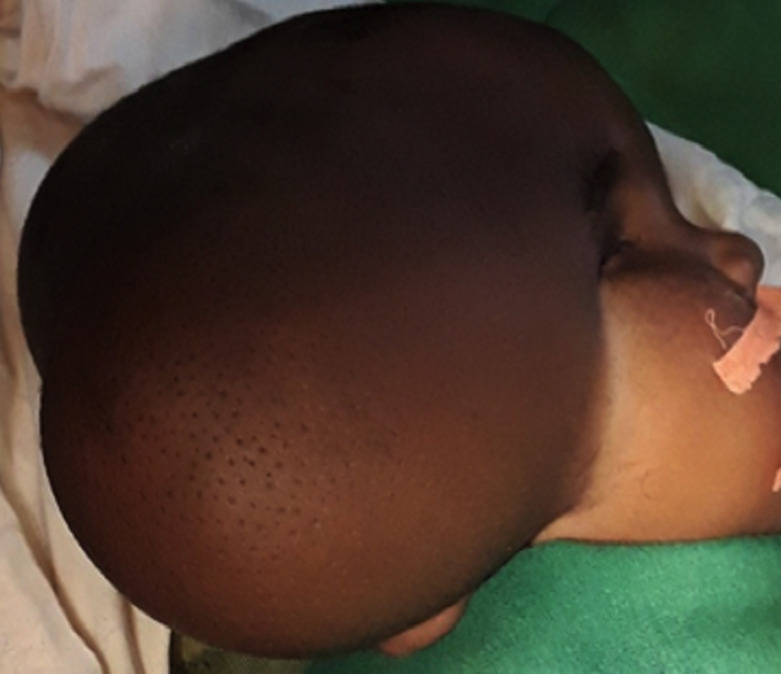
tuméfaction de la région temporo-pariétale droite étendue

**Figure 2:**
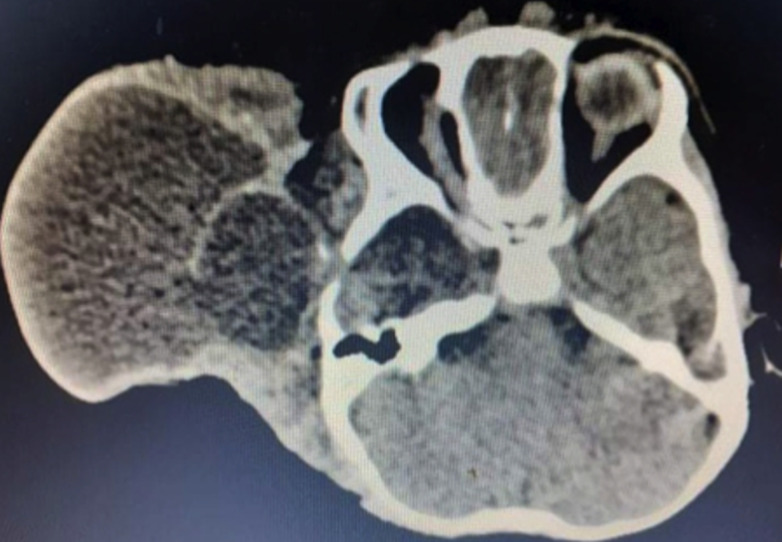
scanner cérébral (coupe axiale) montrant une image d´une méningocèle avec defect osseux temporal droit et une absence du globe oculaire

**Figure 3:**
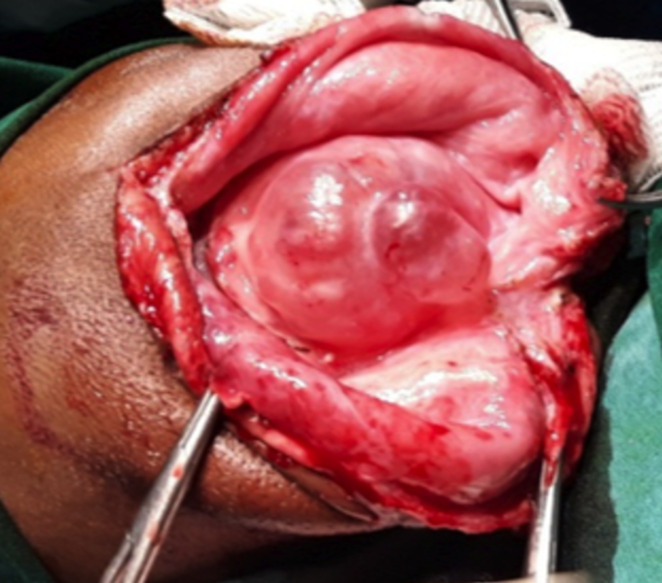
image peropératoire montrant une double couche méningée

**Figure 4:**
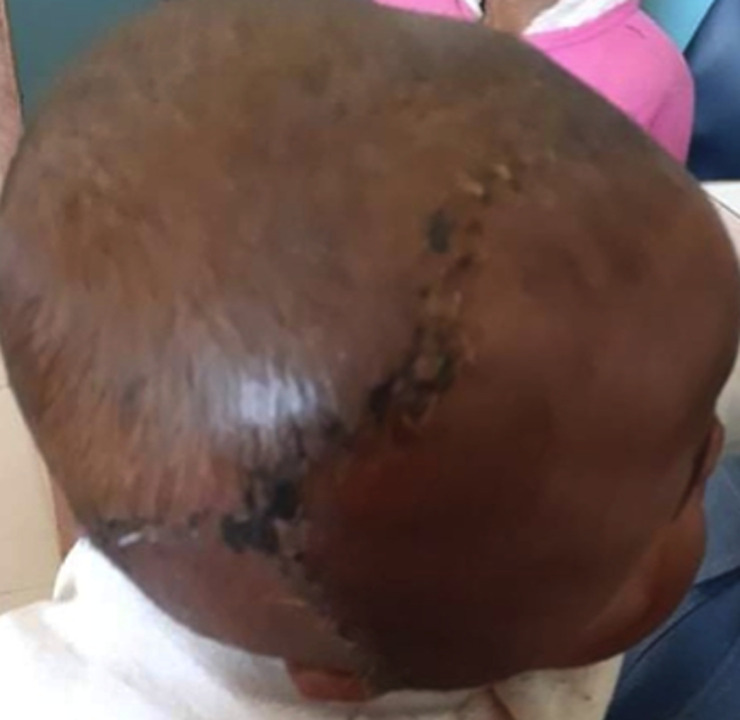
cicatrice à la deuxième semaine post-opératoire

**Figure 5:**
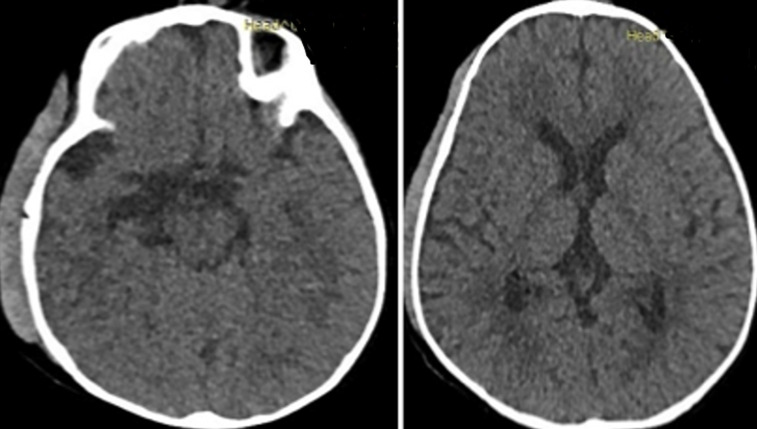
scanner cérébral de contrôle (3 mois post-opératoire) montrant une absence de récidive ni hydrocéphalie

Il s´agissait d´un nourrisson de 12 mois, de genre masculin qui était amené par la famille pour une tuméfaction au niveau de la région temporo-pariétale droite. Il y avait aucun antécédent familial ni périnatal. Les parents constataient la tuméfaction depuis la naissance avec une absence de l´œil droit. La tuméfaction augmentait de volume de façon progressive. A l´examen, on notait une tuméfaction ferme, indolore recouvert d´une peau saine, de dimension 7x10x11cm. La base d´implantation était large (Figure 1). Il y avait une absence d´ouverture de l´œil droit. Le de développement psychomoteur de l´enfant était normal. Il n´y n’avait pas d´autre malformation associée. Le scanner crânien a montré une hernie méningée à travers un défect osseux au niveau de la région temporal droite et une absence du globe oculaire droit (Figure 2). Il a bénéficié d´une intervention neurochirurgicale pour une cure de méningocèle. Au cours de la chirurgie, il y avait une double poche (Figure 3). La suite opératoire était simple. La cicatrice était propre et non inflammatoire (Figure 4). Le scanner cérébral, à 3 mois post-opératoire, montrait une évolution favorable. Il n´y a pas eu une récidive, ni hydrocéphalie (Figure 5).

## Discussion

La méningo-encéphalocèle est près de la ligne médiane par un défaut d´une fermeture du tube neurale. Il peut être au niveau frontal, nasale, nasopharyngé, buccal, naso-orbito-métopique, interpariétal, occipital, sub-oculaire et éthmoïdale [5]. Dans les pays occidentaux, la lésion du crâne concerne l'os occipital dans la ligne médiane postérieure dans 85% des cas. En Asie, la majorité des encéphalocèles sont antérieures et touchent les os frontal ou nasal, mais restent sur la ligne médiane [6]. Dans la littérature, la méningo-encéphalocèle peut être isolée ou associée à d´autre malformation comme: une hydrocéphalie, qui constitue un élément pronostic important [7]; une agénésie du corps calleux, il est le lien entre les deux hémisphères cérébraux [8]; une microcéphalie; des anomalies oculaires (exophtalmie, microphtalmie, cataracte, atrophie choriorétinienne); une malformation cranio-faciale: une fente labio-palatine, une craniosténose, une hypertélorisme avec retrognatsisme; des dysmorphies des membres, des doigts et des orteils; des anomalies des organes génitaux externes. Il y a une élévation de la fréquence des malformations associées dans la méningo-encéphalocèle occipitale par rapport à la localisation antérieure [6]. Concernant notre observation, il s´agit d´une localisation latérale d´une méningocèle au niveau de la région temporale qui est une localisation rare, loin de la ligne médiane. La méningocèle est une malformation rare mais la localisation temporale associe à une malformation oculaire semble exceptionnelle. La malformation oculaire est l´absence du globe oculaire droit. Concernant l´évolution et le pronostic, la suite opératoire a été simple avec cicatrice propre et non inflammatoire. A 3 mois post-opératoire, le scanner cérébral ne montre aucune récidive de la malformation ni hydrocéphalie (Figure 5).

## Conclusion

La méningocèle est une malformation congénitale rare, et la localisation temporale avec malformation oculaire est exceptionnelle. Le scanner crânien permet de poser le diagnostic. Le traitement est chirurgical.
